# Characteristics of Qualitative Research in Behavior**-**Analytic Journals: A Scoping Literature Review

**DOI:** 10.1007/s40617-025-01144-y

**Published:** 2026-02-04

**Authors:** Melanie R Martin Loya, Richard A. Price, Rayan Alqunaysi, Alyssa Barrera-Lansford, Elaine Macias Gilmartin, David Ray G. Miranda, Eric N. Shannon

**Affiliations:** 1 University of California Davis, MIND Institute, Sacramento, CA USA; 2 Educational Studies, Purdue University, West Lafayette, Indiana USA; 3 Department of Special Education, Prince Sattam bin Abdulaziz University, Al-Kharj, Saudi Arabia; 4 University of Texas at Austin, Austin, TX USA; 5 University of Illinois Urbana Champaign, Champaign, IL USA; 6 University of West Georgia, Carrollton, Georgia USA; 7 Rush University Medical Center, Chicago, Illinois USA

**Keywords:** Qualitative, Scoping review, Literature review, Behavior analysis, Methodology

## Abstract

Qualitative research is a common methodology used in fields such as education and medicine, but it is less common in behavior analysis. To explore the current use of qualitative approaches in behavior analysis, we conducted a scoping review aligned with recommendations from Arksey and O’Malley (2005) and Levac et al. (2010) across eight influential behavior-analytic journals to answer the research question: What are the characteristics of qualitative research in behavior-analytic journals? The search and screening resulted in 38 articles meeting the inclusion criteria across five of the eight journals. Data were charted and presented across basic publication metrics, qualitative approaches, study aims, and population or data source information. Most of the included articles were published in *Behavior Analysis in Practice*, utilized multiple methods, involved interviews with caregivers of individuals with disabilities or professionals in applied behavior analysis, and were analyzed using thematic analysis. Recommendations and resources for future qualitative research in behavior analysis are presented.

Qualitative research is an empirical methodology commonly used in fields allied with applied behavior analysis (ABA), such as special education and medicine (Aranda, 2020; Clement et al., 2018; Leko et al., 2021). Qualitative research explores deeper meaning beyond what can be gleaned from a purely quantitative methodological process. Researchers use qualitative methods to highlight people’s lived experiences and capture the meanings they ascribe to those experiences (Patton, 2015). Data collected through qualitative methods resembles natural events, settings, and systems, which helps researchers and consumers of research to better understand the complexity of a given phenomenon (Miles et al., 2014).

Although researchers across all methodologies develop data collection tools and interpret data, qualitative researchers serve as the primary instruments for inquiry in a more immersive way. As such, their positionalities, experiences, and biases directly shape both study designs and the interpretation processes, requiring transparent reporting to support trustworthiness and reflexivity (Miles et al., 2014, Patton, 2015; Tracy, 2020, 2024). These detailed and transparent descriptions of self are critical when examining the role of the researcher within the qualitative research process and the impact their role might have on participant interactions, analyses, and findings (Patton, 2015; Tracy, 2020, 2024). Researcher experiences and biases can be a strength of qualitative research when adequately reported and can enhance the *credibility* and *trustworthiness* of the work, which is analogous to validity and reliability in quantitative research (Miles et al., 2014; Tracy, 2020). To further understand the intricacies of qualitative research, it is essential to consider the philosophical assumptions that underpin this methodology.

## Philosophical Assumptions

Qualitative methodologists have emphasized the philosophical assumptions underlying their research, highlighting that researchers intentionally and unintentionally bring their philosophical assumptions into their studies. These assumptions often influence the theoretical foundations of researchers’ work (Creswell & Poth, 2024). Often, though not universally, qualitative researchers align with the interpretivist or constructivist paradigm, which posits that no single objectively correct reality or knowledge exists. Instead, reality and knowledge are shaped by the personal views, beliefs, and cultural backgrounds of both researchers and the people and phenomena being studied (Tracy, 2024).

In psychology—a field characterized by multiple subdisciplines and diverse theoretical perspectives—quantitative methods rooted in post-positivist assumptions have historically dominated (Willig, 2019). This is particularly evident in experimental psychology, where objectivity, measurement, and causal inference are prioritized (Willig, 2019). In contrast, qualitative research has often been marginalized in certain psychology domains (Brinkmann, 2015; Masaryk & Stainton Rogers, 2024), especially in behavioral psychology, which is the philosophical foundation of ABA (Green, 2015). The emphasis on prediction and control of behavior in behaviorism has led to a preference for quantitative, experimental methodologies (e.g., single-case research) that align with post-positivist assumptions (e.g., objective measurement, systematic manipulation, empirical verification; Baer et al., 1968; Skinner, 1953).

The primary goal of behavioral psychology is to predict and control behavior; therefore, the philosophical assumptions underlying behavioral psychology are designed to support this objective (Skinner, 1987). From a behavioral perspective, achieving this goal requires the application of rigorous scientific methods that collect data systematically, allowing researchers to establish valid, objective conclusions about behavior. This epistemological stance aligns with post-positivism, which acknowledges the possibility of objective knowledge and recognizes that absolute certainty is unattainable. However, qualitative methodology, particularly in its interpretivist form, often assumes that knowledge is multiple and subjective, shaped by the researchers’ and participants’ backgrounds (Willis, 2007).

Despite these differences, some behavioral psychologists have identified areas where behavior analysis and qualitative research may intersect (Roche & Barnes-Holmes, 2003; Stones, 1985; Willig, 2019). Although interpretivism does not align with behaviorism’s primary goal of prediction and control, qualitative research can complement behavioral science by serving purposes beyond prediction, such as understanding contextual variables, subjective experiences, and cultural influences on behavior (Glass, 2007; Hayes et al., 2016; Roche & Barnes-Holmes, 2003). Notably, researchers based in contextual behavioral science, a new form of behavior analysis that has given rise to acceptance and commitment therapy and relational frame theory, have also noted the importance of qualitative research and advancing methodological diversity to enhance research and practice (Jando & Dionne, 2024; Ruan et al., 2023). Indeed, the novelty of qualitative research within behavior analysis creates the opportunity for innovation and the adaptation of knowledge from allied fields for behavior analysis. Some examples of recent innovative uses of qualitative methods include Frost and Ingersoll (2024) and Frost et al. (2021), who employed qualitative methods to help identify the ”active ingredients” of naturalistic developmental behavioral interventions for young autistic children, findings that can be used to optimize evidence-based care.

Qualitative methods are also an important component of mixed methods research (Creswell, 2022; Greene, 2007). Mixed methods has been identified as a promising avenue to bridge research divides in education and disability-serving fields (Snodgrass et al., 2024) and advance and address issues related to diversity, equity, and inclusion of historically marginalized individuals (Leko et al., 2023). In addition, and especially relevant to behavior analysts, the integration of qualitative methods and single case experimental designs is another novel area ripe for further exploration (Onghena et al., 2019). Therefore, exploring the use of qualitative methods within behavior analysis is a prudent step toward ensuring researchers and providers in behavior analysis are up to date with methodological advances and have the tools to be competent consumers and producers of research within and outside of behavior analysis.

Currently, there are a few conceptual articles related to qualitative research in behavior analysis (notable contributions include Burney et al., 2023, 2024; Stones, 1985), and there is an increase in interest in qualitative research, as evidenced by this special issue in *Behavior Analysis in Practice*. Despite this increase in interest, to our knowledge, no literature reviews about qualitative research in behavior analysis have been conducted. To address this gap, we have two aims in this project: (1) To explore the existing uses of qualitative approaches in prominent behavior-analytic journals to enhance our understanding of their scope and characteristics, and (2) To use our results to craft recommendations to improve future qualitative research in behavior analysis.

The research question (RQ) guiding this project was: What are the characteristics of qualitative research in behavior-analytic journals? This pre-registered study (Martin Loya et al., 2024) followed open science practices in alignment with updated quality indicators for literature reviews in the field of special education (Cumming et al., 2023) and recommended guidelines for scoping reviews (Arksey & O’Malley, 2005; Levac et al., 2010). Phases for this study included preparation and training, preliminary search and screening, final search and screening, data charting (i.e., extraction), synthesis, and writing for dissemination.

## Method

To answer our RQ, we conducted a scoping literature review. Scoping reviews are distinct from systematic literature reviews and serve a different purpose. Scoping reviews do not feature quality assessment and are meant to generate a “preliminary assessment of *potential size and scope* of available research literature” (*emphasis added*; Grant & Booth, 2009; p. 101). Scoping reviews are ideal for presenting the landscape of research literature within an unexplored area (Arksey & O’Malley, 2005), and a “qualitative content analysis approach” is a recommended strategy to support the data charting stage (Levac et al., 2010, p. 4), which is like the data extraction phase in systematic literature reviews. Therefore, given the topic area, a scoping review without quality assessment was determined to be the best choice to address our RQ and generate meaningful recommendations for consumers and producers of behavior analytic research.

In addition, it is important to reiterate that this scoping literature review is not meant to represent an exhaustive list of all behavior analytic articles that have used qualitative approaches. Owing to the novelty of qualitative research within the field of behavior analysis, we felt it necessary to (1) Narrow our focus to journals that are entirely behavior analytic in scope and philosophy (i.e., at the exclusion of journals that may have a more education focus, such as the *Journal of Positive Behavior Interventions*, or disability-focused, such as *Autism*), and (2) Limit inclusion to studies that explicitly conceptualized their study as qualitative (i.e., studies that may have used qualitative approaches but never used the word “qualitative,” would not have met criteria).

To prepare for this project and in alignment with recommendations for conducting scoping reviews (Arksey & O’Malley, 2005; Levac et al., 2010), our research team was carefully assembled. Our research team has a range of training and clinical experiences, institutions attended (e.g., our team received doctoral training at three separate universities), and relevant publication experiences. We have also received graduate-level instruction in qualitative inquiry and philosophy, and most team members have experience conducting and publishing qualitative research within and outside the context of behavior analysis (e.g., in special education). All authors are Board Certified Behavior Analysts (BCBAs). While conducting this scoping review, we discussed our biases, strengths, and weaknesses in conducting qualitative work. Our different backgrounds in training and use of qualitative methods were a source of strength that allowed us to explore new insights and understandings that qualitative research can offer and to craft recommendations for the field.

First, our research team collaborated to craft our RQ and then construct and pre-register a scoping review protocol following recommended practices in conducting scoping reviews (Arksey & O’Malley, 2005; Levac et al., 2010) and open-science recommendations (Cumming et al., 2023). Our protocol was updated periodically to reflect all stages and changes in the review process. From our collaborative planning, we decided to limit our search to journals affiliated with the Association for Behavior Analysis International (ABAI) and Society for the Experimental Analysis of Behavior (SEAB), for a total of eight journals. In alignment with other research in behavior analysis (e.g., Cengher & LeBlanc 2024), we chose to limit our inclusion criteria to ABAI and SEAB journals to capture the literature most representative of behavior-analytic research and practice. We believe these journals serve as the primary publication outlets for the field of behavior analysis, which ensures conceptual consistency and alignment with core tenets of behavior analysis. Additionally, this focus also allowed us to maintain a manageable scope and clearly define the boundaries of our review. We recognize that relevant behavior-analytic work can be found in other outlets, but argue that this review represents an initial mapping of the field’s literature, and future reviews may add broader perspectives to enrich the examination of qualitative approaches in behavior-analytic studies.

### Search

ABAI journals included:(1) *The Analysis of Verbal Behavior *(AVB), (2) *Behavior Analysis in Practice *(BAP), (3) *Behavior and Social Issues *(BSI), (4) *Education and Treatment of Children *(ETC), (5)* Perspectives on Behavior Science*(PoBS), (6) *The Psychological Record *(TPR),and SEAB journals included:(7)* Journal of Applied Behavior Analysis* (JABA) and the (8)* Journal of the Experimental Analysis of Behavior *(JEAB)*. *Search procedures were established in consultation with two university librarians who recommended searching directly within each journal’s website rather than using databases. A preliminary search was conducted in June 2024, which was modified and redone in June 2025. The only modification was to streamline the keywords to ensure all relevant articles were identified. All articles that originally met inclusion in the June 2024 search were also included in the 2025 search results, and six additional articles were found to meet inclusion criteria as a result of the 2025 modification. The findings presented in this article reflect the complete 2025 search. The 2025 search only used the keyword “qualitative.” This single keyword was chosen to identify articles that were explicitly conceptualized as qualitative. The search date range was from the earliest publication in each journal to the end of June 2024. The only exception to this was ETC, which did not become an ABAI-affiliated journal until 2020; therefore, the ETC search was from 2020 until the end of June 2024. More detailed search information about the preliminary and final search can be found in our pre-registration protocol (Martin Loya et al., 2024).

### Screening

For screening, inclusion criteria required articles to be:  (1) published in one of the eight approved ABAI or SEAB journals, (2) explicitly conceptualized as qualitative (i.e., the word *qualitative* must have been used to describe the study or approaches used), (3) describe qualitative data analysis in the method section (e.g., “this study used thematic analysis”), and (4) include qualitative findings in the results or findings section (e.g., quotes, photographs, or other non-numeric examples from the data source). Mixed and multiple-method studies were also eligible for inclusion. Exclusion criteria included literature reviews, conceptual or theoretical articles, or purely quantitative articles (i.e., using solely numerical data) without qualitative analysis. The first author initially determined inclusion and exclusion criteria after conducting preliminary searches in each journal to identify effective search and screening strategies. Then, the research team met to discuss and collaboratively decide on the final criteria to best address our RQ. Screening resulted in 38 articles meeting the inclusion criteria for this scoping review. Most articles identified as meeting inclusion criteria were published in BAP (*n* = 16), followed by ETC (*n* = 10), BSI (*n* = 6), JABA (*n* = 4), and TPR (*n* = 2).

#### Reliability, Credibility, and Trustworthiness

**Fig. 1:**
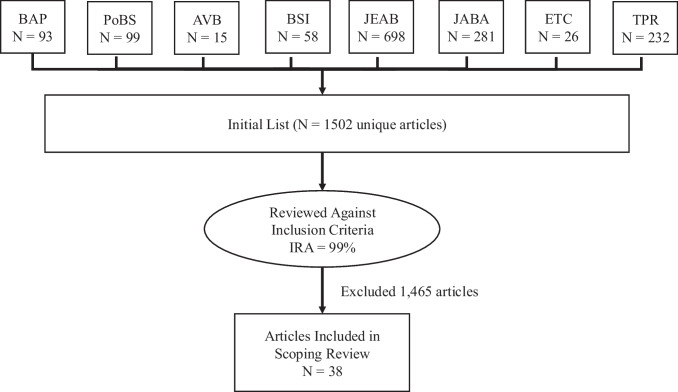
Scoping review search and screening

The first author used an online tool to randomly assign members of the research team to journals and ensured the team members were naïve to the assignments and results of their colleagues. Each team member conducted searches and screenings in at least two different journals. Then, the first author calculated inter-rater reliability (IRR) percentages for the search and screening results between researchers using the following formula: agreements over total number x 100. The search results in all the journals across researchers resulted in 100% IRR. For screening IRR, agreement was high across journals and ranged from 92% to 100%. Overall, reviewers agreed on 1491 of the 1502 articles screened (99% agreement). As mentioned, articles that were included and screened in the preliminary June 2024 search were retained and not re-reviewed, and their original agreement status was incorporated into these calculations. The first author reviewed all disagreements in screening decisions to reach a final decision. See Fig. 1 for a search and screening flow chart.

### Data Charting

Data charting in the present study focused on basic publication metrics (e.g., journal, year published, participant geographic locations), methodology (e.g., type of designs and analyses), reported justifications for using their qualitative analysis method (if any), and population or data source information (e.g., if focused on individuals with disabilities, providers, or caregivers). For methodology, this scoping review classified the article’s qualitative methodology as either  (a) qualitative only, (b) mixed methods (i.e., the authors explicitly stated they used mixed methods, which denotes the purposeful integration of quantitative and qualitative data to arrive at new inferences that would not be possible with a single data source; Johnson et al., 2007), or (c) multiple methods (i.e., authors utilized qualitative methods in conjunction with an additional method, but did not label their work as mixed methods). Additionally, this review considered each article’s qualitative emphasis as either primarily qualitative (i.e., the article had at least one qualitative-based RQ or, when no RQs were present, if the article provided robust evidence of a qualitative focus) or secondarily qualitative (i.e., qualitative approaches were not used to answer an RQ or when qualitative methods were used primarily to support or supplement the primarily quantitative aims). In alignment with the aims of this special issue, we decided that differentiating between primary and secondary qualitative foci could advance our understanding of how and if behavior-analytic researchers are using qualitative approaches that “go beyond social validity.” In this review, using qualitative approaches for social validity without a corresponding RQ or in-depth information about their qualitative approach would result in being coded as secondarily qualitative. When included studies did not have any RQs but provided in-depth information about their qualitative procedures (e.g., a full and detailed paragraph about the qualitative analysis of their social validity data), we coded these studies as having a primary qualitative focus.

To achieve a consensus on the included articles, a collaborative method involving all seven research team members was employed to analyze the final set of included articles and associated data. Each article was randomly assigned, read in full, and charted by at least three research team members. Of note, three articles were purposefully reassigned to avoid potential conflicts of interest and bias because the random assignment resulted in a research team member having a close professional relationship with at least one of the study’s authors. The authors also used note-taking strategies to maintain a log of decisions and promote transparency.

To start the data charting process, identical primary and secondary spreadsheets were created. Then, members of the research team independently reviewed each of their assigned articles and charted relevant data into their respective primary or secondary data charting spreadsheets. Next, the first two authors (who served as the final assessors) reviewed both sets of charted data, article by article, discussing each article and data point until they reached a consensus on the final data to include. A third “consensus” spreadsheet was created through this process. A notable challenge in this process was identifying when thematic analysis (e.g., Braun & Clark, 2012; Ryan & Bernard, 2003) was used. Authors used a wide range of terminology (e.g., “code according to themes” and “analyze thematically”) although often not directly stating that they conducted a thematic analysis nor citing appropriate methodological publications (e.g., citing a popular coding manual rather than published analytical guidance). Given this range and the present lack of guidance in qualitative reporting within behavior analysis, we decided to employ flexible criteria when charting analysis methods to encompass the wide range of sometimes imprecise descriptions. These challenges will be further explored in the “Discussion” section.

Lastly, the data synthesis phase was conducted through writing and collaborating as a team. The first author drafted the initial outline of data synthesis, which was based on the existing data charting sheets. The initial written draft was later reviewed, edited, and expanded upon by the entire research team using an iterative and collaborative writing approach. In addition, we used the recommended qualitative content analysis approach to transform some of the charted data into coherent categories (Levac et al., 2010). For example, to determine Study Aims and General Content, the first three authors collaborated to create four new categories that captured all studies meeting the inclusion criteria (see section below). The data charting and synthesis phases concluded when we felt the data adequately addressed our RQ and provided an accurate and comprehensive written overview of the characteristics of published qualitative research in behavior-analytic journals.

## Results

In this scoping review, we identified 38 empirical articles that employed qualitative approaches and were published in behavior-analytic journals. We outline the results across journal metrics and trends, study aims and general content, and methodological information (e.g., demographics and types of qualitative analyses used).

### Journal Metrics and Trends

**Fig. 2:**
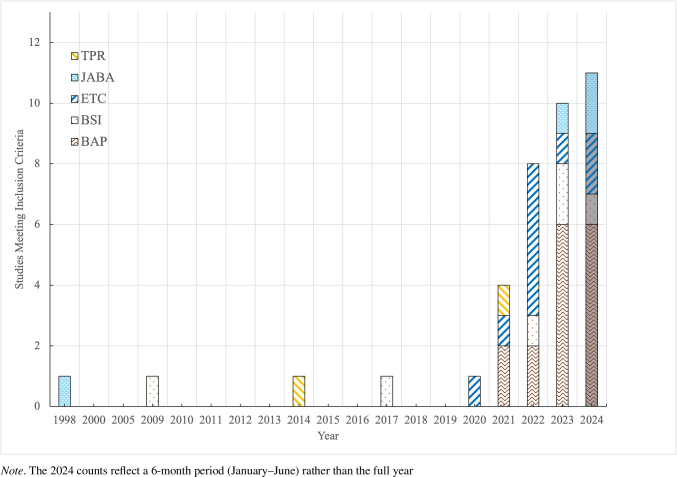
Year and Journal Distribution of Included Studies. *Note.* The 2024 counts reflect a 6-month period (January–June) rather than the full year

Identified studies were published across five of the eight academic journals included in this review. No articles meeting inclusion criteria were found in the JEAB, AVB, or PoBS*.* This was unsurprising because these journals tend to focus on theoretical and experimental research. Almost half of all studies included in this review were published in BAP (*n* = 16). In addition, the included studies were published between 1998 and 2024, with the majority published in 2020 or later (*n* = 34). Notably, the publication of qualitative research in behavior-analytic journals has increased yearly since 2020. One study was published in 2020, four in 2021, eight in 2022, ten in 2023, and 11 qualitative studies were published in behavior-analytic journals from January to the end of June 2024. See Fig. 2 for a bar graph displaying the temporal distribution of articles across journals and see Table 1 for more information.

**Table 1:** Journal Metrics and Studies’ Purposes

Study	Journal	Year	Overarching Study Purpose	Location	Design	Population
Ayvaso et al.	ETC	2024	To explore changes in preservice teachers’ knowledge, confidence, and implementation of naturalistic communication teaching strategies before and after completing an online course	Israel	Multiple	Ed. Pers.
Awasthi et al.	BAP	2021	To describe how an ABA organization in India transitioned services from in-clinic to telehealth	India	Multiple	Caregivers + ABA providers
Beahm et al.	ETC	2021	To investigate the resources educators and other school personnel use to find information on effective behavior management practices	One State	Mixed	Ed. Pers.
Briesch et al.	ETC	2022	To understand the views and needs of leadership regarding universal behavior screening and to identify directions for enhancing resources and professional learning about screening	Mult. States	Multiple	Ed. Pers.
Brown et al.	ETC	2020	To examine how educators’ social and emotional competencies align with their collaborative network	One State	Mixed	Ed. Pers.
Caldarella et al.	ETC	2023	To evaluate a class-wide function-related intervention teams’ implementation in an alternative high school summer program	One State	Multiple	Ed. Pers. + Students
Castro-Hostetler et al.	BSI	2023	To learn about the cultural and language barriers that are faced by Latino families when accessing ABA	One State	Qual	Caregivers
Cengher & LeBlanc	JABA	2024	To conduct a survey of journal editors to develop empirically based guidelines for writing reviews for behavior-analytic journals and inform future training of behavior-analytic scholars	NR	Multiple	Research & Higher Ed. Pers.
Contreras et al.	BAP	2024	To explore the state of philosophy training in graduate behavior analysis training programs and provide a resource	NR	Multiple	Research & Higher Ed. Pers.
D'Agostino et al.	BAP	2023	To examine early intervention providers’ and caregivers’ perceptions and reported use of compassionate care	One State	Mixed	Caregivers + Ed. Pers.
de Carvalho et al.	BSI	2017	To strengthen an empirically based conceptual framework suitable for understanding tagging cultural practices	Brazil	Qual	Other (Taggers)
Dillenburger & McKerr	BSI	2009	To explore the contingencies that older caregivers of children with disabilities face	N. Ireland	Qual	Caregivers
Feil et al.	ETC	2024	To assess the impact of the First Step Next revisions to the First Step program	Mult. States	Multiple	Caregivers + Ed. Pers.
Filter et al.	ETC	2022	To distill, differentiate, and operationally define the Practice Elements and differentially guide frontline implementation of teachers and other expected implementers	One State	Mixed	Research & Higher Ed. Pers.
Fromene & Guerin	TPR	2014	To explore Indigenous Australians who had been labeled as Borderline Personality Disorder views on identity, illness, knowledge, and understanding of mental illness	Australia	Qual	Indiv. w Disab.
Galbally et al.	BSI	2023	To investigate and explore the use of a community-based organization’s efforts to provide free language and literacy screenings for students at risk for language and literacy disabilities	One State	Mixed	Caregivers + Ed. Pers.
Ghai et al.	BAP	2022	To identify the prevalence of ABA clinicians who have incorporated animals into ABA services	Mult. States	Multiple	ABA Providers
Guinness et al.	BAP	2024	To evaluate the social validity of a proposed intervention for caregivers of children with autism	NR	Qual	Caregivers
Heinicke et al.	BAP	2022	To expand Friman’s work by interviewing the top 10 most frequently invited public speakers at major ABA conferences	NR	Qual	ABA Providers
Hughes et al.	JABA	1998	To identify and validate critical components of high school students’ conversational behavior that could serve as instructional targets for teaching secondary students with [intellectual disability]	NR	Multiple	Ed. Pers. + Students + Indiv. w Disab. + Other (Employers)
Huscroft‐D’Angelo et al.	ETC	2022	To explore education professionals' perspectives on the preparedness, educational needs, and support systems for students transitioning from foster care to permanency placements	One State	Multiple	Ed. Pers.
Kelly et al.	BAP	2023	To explore how behavior analysts identify ethical dilemmas and make ethical decisions	NR	Qual	ABA Providers
Kranak et al.	BAP	2023	To understand the behaviors of BCBAs when they engage in professional development activities	NR	Multiple	ABA Providers
Lee et al.	ETC	2022	To describe the development of the online modules for parents of young children who exhibit challenging behaviors and to describe the acceptability and feasibility of the modules	Mult. States	Multiple	Caregivers
Liddon et al.	BAP	2024	To collect information on how qualified supervisors are currently providing unrestricted learning opportunities for their trainees	Mult. Countries	Multiple	ABA Providers
Marchese & Weiss	BAP	2023	To develop and evaluate an assessment tool (i.e., the Parent Partnership Questionnaire)	NR	Multiple	Caregivers
Martin Loya & Meadan	BAP	2024	To understand the challenges and needs of U.S.-based Spanish-speaking behavior analysts who work with young children with autism and their families	Mult. States	Qual	ABA Providers
Mayo & Hofmann	BAP	2024	To analyze and summarize ABA in Vermont	One State	Multiple	ABA Providers
Mead Jasperse et al.	BAP	2023	To investigate behavior-analytic researchers use of consent and assent	NR	Multiple	Research & Higher Ed. Pers.
Morris et al.	BAP	2023	To determine why and how clinicians change reinforcers, replicate Graff and Karsten’s (2012) methods, and evaluate how different factors influence practices	Mult. Countries	Multiple	ABA Providers
Moya et al.	ETC	2022	To evaluate student, staff, and parent perceptions of the wellness center at a Utah high school	One State	Multiple	Caregivers + Students + Ed. Pers.
Price et al.	BAP	2025	To explore how individuals interact in subreddits of ABA Reddit in- or out-of-alignment with the 2022 Ethics Code	Other	Qual	Other (Social Media)
Schena et al.	JABA	2024	To examine the comparative efficacy and social validity of two instructional components on student test performance in an undergraduate course	One State	Multiple	Postsecondary
Simmons et al.	BAP	2021	To examine the acceptability and feasibility of virtual supervision for BCBA/BCaBA trainees during the COVID-19 pandemic	Mult. States	Multiple	Postsecondary
St Peter et al.	JABA	2023	To explore the reasons for the infrequent reporting of procedural fidelity data in behavior-analytic studies and to understand the perspectives of scholars in applied behavior analysis	NR	Qual	Research & Higher Ed. Pers.
Suarez-Balcazar et al.	BSI	2022	*Study 1:* To explore goal setting on achieving health-related behaviors of Latinx parents of children with disabilities *Study 2:* To increase caregivers of children with ASD knowledge and practice of puberty and sexuality	Mult. Countries	Qual	Caregivers
Taira & Maunakea	BSI	2023	To explore culturally responsive educational practices of five educators in Hawaiʻi	One State	Qual	Research & Higher Ed. Pers.
Tyrberg et al.	TPR	2021	To assess the feasibility of the Wisconsin Card Sorting Test and to explore the verbal behaviors and relational frames involved in executive functioning as measured by the test	Sweden	Multiple	Other Providers

*Note*: In the Journal column, *BAP* Behavior Analysis in Practice, *ETC* Education and Treatment of Children, *TPR* The Psychological Record, *JABA* Journal of Applied Behavior Analysis, and *BSI* Behavior and Social Issues. In the Locations column, *One State* one U.S. state, *Mult. States* multiple U.S. states, *Mult. Countries* multiple countries. In the Design column, *Multiple* multiple methods, *Mixed* mixed methods, *Qual* qualitative methods (only). In the Population column, *Postsecondary* Postsecondary Individuals, *Caregivers* caregivers of individuals with disabilities, *Ed.* means education, *Pers.* personnel, *Indiv. w Disab.* Individuals with Disabilities, *Students* children in primary education settings

### Study Aims and General Content

To generate categories, the research team utilized a content analysis approach to summarize and synthesize studies’ RQ(s) and their purpose statements to create concise descriptions that adequately captured the studies’ aims, using researchers’ own words whenever possible. However, 14 of the included studies did not report any RQs, so we collaboratively generated our own concise descriptions based on their reported procedures when existing ones were not available. Specifically, in the data charting phase, three researchers independently wrote concise aims based on available data, which were synthesized and finalized by the first author. The four categories generated include: (1) Exploring How Professionals and Families Navigate Services, (2) Evaluating Interventions, Social Validity, Delivery Models, and Program Implementation, (3) Cultural and Contextual Factors in Services, and (4) Developing Tools and Frameworks. One study did not fit within any categories and has been identified as “other” (Price et al., 2025).

Price et al. (2024) conducted a content analysis of Reddit posts in ABA-related forums to assess if user content was aligned or misaligned with the Behavior Analyst Certification Board (BACB) Ethics Code. The four categories are further described below, and additional details about each study can be found in the Table 1 column titled *Overarching Study Purpose*.

#### Exploring How Professionals and Families Navigate Services

Fourteen studies were identified under this category. Most authors utilized multiple methods to address their research aims (*n* = 7), followed by mixed methods (*n* = 4), and qualitative only (*n* = 3). Articles in this category explored a range of questions related to navigating services, such as the prevalence and perceptions of ABA clinicians incorporating animals into services (Ghai et al., 2022), BCBAs’ continuing education activities (Kranak et al., 2023), and an exploration of the experiences of older caregivers of children with disabilities (Dillenburger & McKerr, 2009).

#### Evaluating Interventions, Social Validity, Delivery Models, and Program Implementation

Thirteen studies were identified under this category. Most authors used multiple methods (*n* = 11), followed by mixed methods (*n* = 1) and qualitative-only methods (*n* = 1). Several studies explored the feasibility and acceptability of programs serving populations in public schools (e.g., Caldarella et al., 2023; Filter et al., 2022; Moya et al., 2022), and others examined aspects of clinical practice, such as program implementation (Briesch et al., 2022), impact of telehealth supervision on BCBAs (Simmons et al., 2021), or students’ and parents’ skill acquisition (Awasthi et al., 2021; Lee et al., 2022).

#### Cultural and Contextual Factors in Services

Five articles were identified under this category. The majority of articles utilized only qualitative methods to address their research aims (*n* = 4), and one used multiple methods (Castro-Hostetler et al., 2023). Three of the articles examined various aspects of developmental disability services for Hispanic or Latino/x populations, such as provider experiences (Martin Loya & Meadan, 2024), service access barriers (Castro-Hostetler et al., 2023), or exploring the impacts of services on caregivers (Suarez-Balcazar et al., 2022). In addition, two studies explored Indigenous experiences in education and disability care; for example, educators in Hawaiʻi shared experiences with culturally responsive teaching (Taira & Maunakea, 2023), and Indigenous Australians shared perspectives on their identities and mental health (Fromene & Guerin, 2014).

#### Developing Tools and Frameworks

Five articles were identified in this category. Three studies reported using multiple methods (Cengher & LeBlanc, 2024; Contreras et al., 2024; Marchese & Weiss, 2023), and two articles employed qualitative methods only (de Carvalho et al., 2017; Heinicke et al., 2022). Four studies developed tools to support professionals, such as Heinicke et al. (2022), who interviewed frequently invited speakers at behavior-analytic conferences to develop a checklist for presenters, and Marchese and Weiss (2023), who developed and evaluated a questionnaire to assist clinicians in developing a positive rapport and partnership with parents. The final study included in this category was unique in behavior-analytic literature; de Carvalho et al. (2017) conducted an ethnographic study to explore variables controlling cultural behaviors in Brazil (i.e., tagging). They then used their findings to propose a behavior-analytic framework to replace tagging with alternative “prosocial” behaviors.

### Methodological Information

Despite the focus on qualitative research, the included studies were methodologically wide-ranging. We charted study designs and qualitative emphasis, data and population sources, data collection strategies, analyses, and reported justifications for their analysis methods. These data are further outlined below and summarized in Tables 1 and 2.

**Table 2:** Methodological information and reporting

Study	Qual Emphasis	Qual Method		Qual Analyses		
		*Type*	*Multi, other*	*Type*	*Multi, other*	*Justification*
Ayvaso et al.	Primary	Other	Observational notes	NR	–	Enhance understanding
Awasthi et al.	Primary	Interviews	–	NR	–	NR
Beahm et al.	Primary	Focus groups	–	NR	–	Enhance understanding
Briesch et al.	Secondary	Open-ended	–	Thematic	–	NR
Brown et al.	Primary	Interviews	–	Other	LaRossa's (2005) three-phase analysis	Complement quantitative
Caldarella et al.	Secondary	Multiple	Interviews, open-ended	NR	–	NR
Castro–Hostetler et al.	Primary	Multiple	Open–ended, Interviews, focus groups	Multiple	Thematic, in –vivo coding	ID themes
Cengher & LeBlanc	Primary	Open–ended	-	Thematic	-	Enhance understanding
Contreras et al.	Secondary	Open–ended	-	Thematic	(conducted with A.I.)	NR
D'Agostino et al.	Primary	Multiple	Open-ended, care logs, interviews	NR	–	Complement quantitative
de Carvalho et al.	Primary	Multiple	participative naturalistic Observation, Interviews	Other	Behavior systems analysis	Theory testing
Dillenburger & McKerr	Primary	Interviews	–	Other	IPA	Theory testing
Feil et al.	Primary	Focus groups	–	CCM	–	NR
Filter et al.	Primary	Multiple	E-Delphi, content analysis	Content analysis	–	ID themes
Fromene & Guerin	Primary	Other	Indigenous	Indigenous	–	Enhance understanding
Galbally et al.	Primary	Interviews	–	Thematic	–	NR
Ghai et al.	Secondary	Open-ended	–	Thematic	–	NR
Guinness et al.	Primary	Interviews	–	Thematic	–	NR
Heinicke et al.	Primary	Interviews	–	Thematic	–	NR
Hughes et al.	Secondary	Interviews	–	CCM	–	Enhance understanding
Huscroft‐D’Angelo et al.	Primary	Focus groups	–	Thematic	–	ID themes
Kelly et al.	Primary	Interviews	–	Thematic	–	Report experiences
Kranak et al.	Secondary	Open-ended	–	Other	Phenomenological/basic qualitative	Report experiences
Lee et al.	Secondary	Interviews	–	NR	–	Report experiences
Liddon et al.	Secondary	Open-ended	–	NR	–	NR
Marchese & Weiss	Primary	Interviews	–	NR	–	NR
Martin Loya & Meadan	Primary	Interviews	–	Thematic	–	Enhance understanding
Mayo & Hofmann	Primary	Open-ended	–	Thematic	–	Enhance understanding
Mead Jasperse et al.	Primary	Open-ended	–	NR	–	NR
Morris et al.	Primary	Open-ended	–	NR	–	NR
Moya et al.	Secondary	Open-ended	–	Other	Grounded theory	NR
Price et al.	Primary	Other	Content analysis	Content analysis	–	Enhance understanding
Schena et al.	Secondary	Open-ended	–	NR	–	NR
Simmons et al.	Primary	Open-ended	–	CCM	–	NR
St Peter et al.	Primary	Focus groups	–	Thematic	–	ID themes
Suarez-Balcazar et al.	Primary	Interviews	–	Content analysis	–	NR
Taira & Maunakea	Primary	Other	Indigenous storytelling	Indigenous	–	Enhance understanding
Tyrberg et al.	Primary	Interviews	–	RFT analysis	–	NR

*Note*: In the Qual Analysis columns*,* and *Thematic* Thematic Analysis. In the Qual Analysis column, *Type*, *CCM* Constant Comparative Method, and *RFT* Relational Frame Theory. In the Qual Analysis column, *Multi, Other, IPA* Interpretative Phenomenological Analysis. In the Qual Analysis column, *Justification, ID Themes* Identify Themes. *NR* Not Reported

#### Study Designs and Qualitative Emphasis

Studies included in this review mostly used multiple research methods (*n* = 21), followed by qualitative-only (*n* = 12), and a few studies used mixed methods (*n* = 5). In addition, most (*n* = 28) of the included studies were identified as having a primary qualitative emphasis, and ten were identified as having a secondary qualitative emphasis. Some of the studies with a secondary qualitative emphasis reported minimal information related to their qualitative methods or findings (e.g., Liddon et al., 2024; Kranak et al., 2023; Ghai et al., 2022; Moya & Hofmann, 2024), and other studies used an experimental design and the qualitative portions were solely for social validity purposes and not explored in-depth (e.g., Schena et al., 2024; Caldarella et al., 2023).

### Data and Population Sources

Most of the included studies were conducted solely in the United States (*n* = 18), the majority of which were within a single US state (*n* = 12), and fewer were conducted across multiple states (*n* = 6). Two studies were conducted in Europe (i.e., N. Ireland and Sweden; Dillenburger & McKerr, 2009; Tyrberg et al., 2021), one in Australia (Fromene & Guerin, 2014), one in Brazil (de Carvalho et al., 2017), one in India (Awasthi et al., 2021), one in Israel (Ayvaso et al., 2024), and three studies were conducted across multiple countries (Liddon et al., 2024; Morris et al., 2023; Suarez-Balcazar et al., 2022). For example, Suarez-Balcazar et al. (2022) conducted case studies in the US and Colombia, and both Liddon et al. (2024) and Morris et al. (2023) gathered survey results from participants across seven and six countries, respectively (e.g., US, Canada, Italy, United Kingdom, United Arab Emirates). Ten studies did not explicitly report geographic data (e.g., St. Peter et al., 2023; Mead Jasperse et al., 2023) or reported nonspecific geographic information (e.g., “North America”; Marchese & Weiss, 2023), and one study did not have human participants (i.e., they analyzed anonymous online behavioral products) and thus had no geographical information to report (Price et al., 2025).

The populations or data sources varied across the studies and have been sorted across eight categories: (1) Caregivers of Individuals with Disabilities (*n* = 11); (2) ABA Providers (e.g., BACB certificants; *n* = 9); (3) Early and Secondary Education Personnel (e.g., Early intervention providers, special education teachers; *n* = 10); (4) Researchers and Higher Education Personnel (e.g., Researchers who study disability, university professors; *n* = 6); (5) Postsecondary Individuals (i.e., undergraduates, those seeking BACB certification; *n* = 2); (6) Other Providers (e.g., community partners, psychiatric clinical staff; *n* = 2); (7) Individuals with Disabilities (*n* = 2); and (8) Other (i.e., Taggers, Social Media posts; *n* = 2). The numbers do not equal 38 because seven studies reported multiple populations across different categories.

### Data Collection Strategies

Data collection strategies varied. Out of the 38 empirical articles, the most common strategies were to conduct interviews (*n* = 17; e.g., Awasthi et al., 2021; Hughes et al., 1998), followed by open-ended survey questions (*n* = 15; e.g., Briesch et al., 2022; Cengher & LeBlanc, 2024; Kranak et al., 2023). Researchers also used focus groups (*n* = 5; e.g., Beahm et al., 2021; Feil et al., 2024), indigenous talking or storytelling (*n* = 2; Fromene & Guerin, 2014; Taira & Maunakea, 2023), content analysis (Price et al., 2025), and observational notes (Ayvazo et al., 2024). The numbers do not equal 38, because five studies used more than one qualitative strategy. For example, de Carvalho et al. (2017) used two strategies (i.e., participative naturalistic observations and interviews), and D’Agostino et al. (2023) used three different qualitative strategies (open-ended survey questions, care logs, and interviews) to answer their research questions.

### Analyses Methods and Reported Justifications

Many studies did not report their method of analysis nor provide information beyond stating that a “qualitative” analysis was conducted (*n* = 11). The most commonly reported analysis method was thematic analysis (*n* = 13; e.g., Contreras et al., 2024; Guinness et al., 2024). Some studies also reported using content analysis (*n* = 3; Filter et al., 2022; Price et al., 2025; Suarez-Balcazar et al., 2022), constant comparison method (*n* = 3; Feil et al., 2024; Simmons et al., 2021), and Indigenous methods of analysis (*n* = 2; Fromene & Guerin, 2014; Taira & Maunakea, 2023). Seven studies reported other methods of analysis, such as elemental in vivo coding (Castro-Hostetler et al., 2023), behavior systems analysis (de Carvalho et al., 2017), analyses based on relational frame theory (Tyberg et al., 2021), phenomenological/basic qualitative approach (Kranak et al., 2023), interpretative phenomenological analysis (IPA; Dillenberger & McKerr, 2009), LaRossa’s (2005) three-phase analysis (Brown et al., 2020), and grounded theory (Moya & Hoffmann, 2022). The reported *n* in this section does not equal 38 because one study used more than one method of analysis (Castro-Hostetler et al., 2023).

In addition, nearly half (*n* = 18) of the included articles did not provide concrete justifications for the analysis methods used. Of the half that did justify, most reported their justification was to enhance understanding (*n* = 11; e.g., Beahm et al., 2021; Fromene & Guerin, 2014). For example, Beahm et al. (2021) reported, “We collected qualitative data from focus groups with a subgroup of survey participants to deepen our understanding of the survey results’ (p. 203). Other studies reported practical justifications such as to identify themes (*n* = 4; e.g., Castro-Hostetler et al., 2023; Filter et al., 2022; Huscroft‐D’Angelo et al., 2022) or to report participants’ experiences (*n* = 3; Kelly et al., 2023; Kranak et al., 2023; Lee et al., 2022). Finally, others aimed to test theory (*n* = 2; de Carvalho et al., 2017; Dillenberger & McKerr, 2009), for example, Dillenberger and McKerr (2009) reported, “IPA is an inductive, bottom-up methodology in which experiential accounts can be related to wider theoretical contexts. As such, the experiential relevance or adequacy of a given theory can be examined” (p. 159).

## Discussion

This study aimed to explore the characteristics of qualitative research in behavior-analytic journals. Eight journals were searched for qualitative work, yielding 38 articles across five journals meeting the inclusion criteria, with three journals having no published qualitative articles. Results indicated a clear increasing trend in the publication of qualitative research in behavior-analytic journals, particularly in *BAP*. Included studies explored various topics but mostly involved US-based caregivers of individuals with disabilities or ABA providers and explored how professionals and families navigate services. Methodologically, most studies utilized multiple research methods (e.g., a survey analyzed both quantitatively and qualitatively), interviews as their data collection strategy, and thematic analysis as their primary form of qualitative analysis. In addition, across all charted data, missing, incomplete, or inconsistently reported qualitative information was widespread (e.g., 14 studies did not report any RQs and nearly half did not report a justification for their method of qualitative analysis). Therefore, our results indicate that although qualitative research is increasing in behavior-analytic journals, guidance is needed to conduct and report qualitative work in a behavioral context. Next, we will outline study limitations and implications for researchers in ABA.

### Limitations

All research, including the present study, has limitations that need to be considered. First, our search strategy was not all-encompassing, and it is likely that studies using qualitative strategies were not included due to missing keywords. For example, if researchers utilized focus groups and thematic analysis, but did not use the word *qualitative* in their article, it would not have appeared in our search results, nor would it have met the inclusion criteria. In addition, this scoping review was purposefully narrow and included only ABAI and SEAB journals. Other journals may have published qualitative research related to behavior analysis that was not included in this scoping review. Although no literature review is perfect, we believe our methods were successful in capturing the general landscape of qualitative research published in prominent behavior-analytic journals from their inception up to June 2024 and that we have laid a groundwork for further exploration.

### Implications

This study offers significant implications for researchers in ABA. Our results highlight the primary takeaway: There is a great opportunity for improvement and exploration of qualitative research in behavior analysis.

#### Drawing on Existing Frameworks and Guidelines

As noted in the introduction, qualitative research has a long history in the social sciences, including related fields such as psychology (Willig, 2019) and special education (Leko et al., 2021); therefore, numerous guidelines and quality indicators for qualitative research are readily accessible. Because most behavior analysts work with autistic children (BACB, n.d.), the field of special education is an excellent starting point. The Council for Exceptional Children (CEC) is a leading international organization for professionals (e.g., special educators) who support children with disabilities. The CEC has published two iterations of quality indicators for qualitative research, the first of which was published in 2005 by Brantlinger et al., which remains invaluable. A group of qualitative researchers in special education published an updated version (The QR Collective et al., 2023), building upon Brantlinger et al.’s (2005) work to enhance quality indicators that guide future research toward improving equity and open-science practices in qualitative studies. We recommend that behavior-analytic researchers and practitioners read these open-access articles to familiarize themselves with qualitative quality indicators.

Additionally, engaging in self-learning and seeking mentorship in qualitative methods can help address some of the challenges observed in the studies included in our review. For instance, many studies referred to thematic analysis procedures while citing Saldaña (2021), a well-known coding manual not intended to replace theoretical frameworks and not exclusive to thematic analyses. In the opening chapter, Saldaña states, “This manual serves as a reference to *supplement* existing works in qualitative research design and fieldwork” (emphasis added; 2017, p. 4). We believe this may arise from misunderstandings regarding qualitative methods or strategies compared to methodologies, which could contribute to inadequate reporting (see more below). For example, all the included studies used at least one qualitative method (e.g., a data collection strategy such as conducting interviews), but most did not fully report relevant methodologies (e.g., detailing theoretical underpinnings or justifying their analytical methods).

#### Strengthening Reviewer and Editorial Practices

Editorial practices are an additional factor that influences the process of publishing research, such as restrictive page limits or inadequately prepared reviewers. Qualitative researchers have reported challenges in the peer review process, including reviewers lacking sufficient expertise and experience in qualitative research, along with a tendency for reviewers to request the quantification of qualitative work (e.g., indicating precisely how many participants expressed views aligned with each theme; Clarke et al., 2025). Anecdotally, our research team has encountered similar challenges when seeking to publish qualitative research in behavior-analytic and other types of peer-reviewed journals. Despite these hurdles, we believe qualitative research has a valid place and future within behavior analysis.

To address these challenges, we believe editorial preparation is important for advancing qualitative research in behavior-analytic journals. Although the development of formal reviewer training is beyond the scope of this paper, professional organizations, journal editors, and board members might consider developing and offering resources to help reviewers engage effectively with qualitative submissions. In consideration of our finding that the majority of qualitative work published in behavior-analytic journals was done so within a multiple methods design (e.g., an experimental design and a qualitative component), we recommend that editors take care to recruit reviewers experienced in qualitative research and not only focus on the primary methodologies or content areas. This may require editors to expand their reviewer recruitment protocols and enlist the support and expertise of early-career or non-behavior-analytic researchers as needed. Doing so can enhance the visibility of high-quality qualitative research in behavior-analytic journals, thereby improving readers’ knowledge and familiarity with qualitative research in behavior analysis.

#### Enhancing Reporting and Reflexivity

Another critical takeaway from our findings is the necessity for improved reporting. Just as in quantitative research, qualitative research offers a variety of standards and reporting guidelines that researchers can select and adapt to best meet their objectives. We found that many studies did not report fundamental information typically required in any empirical study, such as research questions, geographical information, or detailed enough reporting to facilitate critical consumption of research. Although qualitative research is not intended to be generalizable, detailed reporting is still necessary to promote trustworthiness and credibility. For instance, it proved particularly challenging for our research team to identify which studies employed thematic analysis because the descriptions varied significantly, and foundational works in thematic analysis were frequently not cited (e.g., Braun & Clarke, 2012). We encountered numerous descriptions of “thematic analysis” (e.g., “coding for themes,” “analyzing thematically”), prompting us to expand our data charting protocol to include the diverse descriptions observed. However, we acknowledge that “thematic analysis” is often misapplied across various fields (Braun & Clarke, 2023, 2024a), and this challenge is not exclusive to behavior-analytic researchers. Anecdotally, we noted a lack of positionality statements were reported among the included studies; however, we did not systematically assess quality indicators. As mentioned in the introduction, researchers’ experiences and biases directly influence qualitative data collection and analysis and thus are an important consideration when reporting qualitative research that may improve credibility and trustworthiness, similar to how validity and reliability function in quantitative research (Brantlinger et al., 2005; Miles et al., 2014; Tracy, 2020). More information and recommendations for writing positionality statements (also known as reflexivity statements) can be found in allied fields such as rehabilitation psychology (Lund et al., 2025) and school psychology (Sabnis & Wolgemuth, 2023).

As stated, several tools are available from allied fields that behavior-analytic researchers can reference and adapt to their needs, including reporting guidelines such as the Consolidated Criteria for Reporting Qualitative Research (Tong et al., 2007) and the Standards for Reporting Qualitative Research (O’Brien et al., 2014). However, we encourage researchers to exercise judgment when choosing tools, as they are not one-size-fits-all (e.g., Braun & Clarke, 2024b). We also acknowledge, and it is apparent from this review, that there is no singular correct way to conduct qualitative research; however, many tools and quality indicators exist to assist researchers in promoting credibility and trustworthiness in their work, which can improve the overall rigor of qualitative research in behavior analysis (Brantlinger et al., 2005; The QR Collective et al., 2023). In the years following this special issue, and once researchers in behavior analysis have had the opportunity to learn more about qualitative research, we suggest that researchers undertake a systematic literature review focusing on the rigor of the included studies, which would be beneficial in further analyzing and commenting on credibility and trustworthiness.

#### Conceptual Bridges Within Behavior Analysis

We acknowledge that incorporating qualitative strategies may seem antithetical to behavior analysis to some researchers, and they may be deterred from learning about or incorporating qualitative approaches. We suggest that learning about new waves of behavior analysis (e.g., contextual behavior science) might offer a bridge for traditionally trained behavior analysts (Jando & Dionne, 2024; Ruan et al., 2023). As Jando and Dionne (2024) argue, contextual behavior science embraces methodological pluralism but is grounded in behavioral principles. Learning about relational frame theory and acceptance and commitment therapy, and viewing qualitative research as complementary, may help with the adoption and understanding of qualitative approaches. We believe expanding our field’s methodological repertoire does not require a full departure from behavioral principles and philosophy and can offer greater insights into contextual factors impacting those implementing and receiving care based on behavior analysis.

## Conclusion

Historically, qualitative research in behavior-analytic journals has been scarce, but as evidenced in the present review, researchers have been increasingly employing qualitative strategies, which we suspect will continue to rise with the publication of the present special issue in BAP. Consequently, behavior-analytic researchers will need more support to understand qualitative research methods, analyze findings, and adhere to best practices and guidelines. This support should include guidance on the theoretical foundations of qualitative research compared to behavioral paradigms and developing new editorial guidelines for qualitative submissions. Our goal was to paint the current landscape of qualitative research published in prominent behavior-analytic journals and provide recommendations for future research and practice, and to encourage current and future behavior-analytic researchers to strengthen the meaningful and rigorous use of qualitative research in ABA.

## Data Availability

The data that support the findings of this study are openly available at: 10.17605/OSF.IO/SZ6GM
